# A 16-Year Study on Incidence and Progression of Diseased Sphenoethmoidal (Onodi) Cell

**DOI:** 10.1155/2020/2410415

**Published:** 2020-06-05

**Authors:** Ethan I. Huang, Chia-Ling Kuo, Li-Wen Lee

**Affiliations:** ^1^Department of Otolaryngology, Chang Gung Memorial Hospital, Chiayi, Taiwan; ^2^School of Medicine, Chang Gung University, Taoyuan, Taiwan; ^3^Connecticut Institute for Clinical and Translational Science, University of Connecticut Health Center, Farmington, Connecticut, USA; ^4^Department of Community Medicine & Health Care, University of Connecticut Health Center, Farmington, Connecticut, USA; ^5^Department of Diagnostic Radiology, Chang Gung Memorial Hospital, Chiayi, Taiwan; ^6^Department of Nursing, Chang Gung University of Science and Technology, Chiayi Campus, Taiwan

## Abstract

Traumatic operative injury of the optic nerve in an endoscopic sinus surgery may cause immediate or delayed blindness. It should be cautioned when operating in a sphenoethmoidal cell, or known as Onodi cell, with contact or bulge of the optic canal. It remains unclear how frequent progression to visual loss occurs and how long it progresses to visual loss because of a diseased sphenoethmoidal cell. Research to discuss these questions is expected to help decision making to treat diseased sphenoethmoidal cells. From July 2001 to June 2017, 216 patients received conservative endoscopic sinus surgery without opening a diseased sphenoethmoidal cell. We used their computed tomography images of paranasal sinuses to identify diseased sphenoethmoidal cells that could be associated with progression to visual loss. Among the 216 patients, 52.3% had at least one sphenoethmoidal cell, and 14.8% developed at least one diseased sphenoethmoidal cell. One patient developed acute visual loss 4412 days after the first computed tomography. Our results show that over half of the patients have a sphenoethmoidal cell but suggest a rare incidence of a diseased sphenoethmoidal cell progressing to visual loss during the follow-up period.

## 1. Introduction

Severe visual loss is a major complication of endoscopic sinus surgery (ESS) (e.g., see [1]) when damaging to the optic nerve in the sphenoid sinus or more commonly in a sphenoethmoidal cell. A sphenoethmoidal cell or Onodi cell [2], the posteriormost ethmoidal air cell that extends posteriorly to lie superolateral to the sphenoid sinus, has been listed as a high-risk area in ESS (e.g., see [3]). The optic canal may swell or protrude outward a sphenoethmoidal cell and be recognized as an air cell during the procedures. Traumatic operative injury to the optic nerve may cause immediate or delayed blindness [4].

The disease in the sphenoethmoidal cell itself can lead to visual loss. The progression to visual loss is affected by numerous factors, including characteristics and location of the disease. Optic neuropathy or orbital apex syndrome has been reported to be caused by diseased sphenoethmoidal cells, such as an isolated mucocele in a lateral sphenoethmoidal cell [5–7], a mucocele in a central sphenoethmoidal cell [8–10], multiple mucoceles in ethmoid cells [11], sphenoethmoidal cell polyps or sinusitis [12, 13], or a fungal infection within a sphenoethmoidal cell [14]. Another factor is surgical or nonsurgical intervention. Nonsurgical resolution has been reported in an anterior clinoidal mucocele causing optic neuropathy—the visual acuity recovered 1 month after the onset with oral antibiotic treatment [15]. The likelihood of visual loss and the progression time remain unclear and require research that will help decision making to treat diseased sphenoethmoidal cells.

This study is aimed at identifying incidence and location of the sphenoethmoidal cell in a relatively large number of patients undergoing conservative ESS. The study also is aimed at presenting the relationship among the incidence of an existing sphenoethmoidal cell, the incidence of a diseased one, and visual loss.

## 2. Materials and Methods

From July 2001 to June 2017, we enrolled patients who met these criteria:

- With at least one of computed tomography (CT) of paranasal sinuses
- Underwent an ESS performed by Huang
- Without a diseased sphenoethmoidal cell opened unless there were visual symptoms

We identified a sphenoethmoidal cell and its disease status in CT scans ordered to evaluate a patient's first or recurrent chronic rhinosinusitis, based on axial and coronal planes with help of other information from sagittal planes as recommended by Chmielik and Chmielik [16]. The CT image was in a bone window setting to optimally display thin air-cell bones. The risk of progression to visual loss was assessed by any diseased sphenoethmoidal cells. We defined a diseased lateral sphenoethmoidal cell as the opaque posteriormost ethmoidal air cell that extends more than or equal to 5 mm posteriorly to lie superolateral to the sphenoid sinus and contacts or encases an optic canal. We defined a diseased central sphenoethmoidal cell as an opaque posterior ethmoid cell that lies superiorly and extends more than or equal to 5 mm to the sphenoid sinus in a midline position, contacting or encasing at least one optic canal. We counted the patient having an opaque sphenoethmoidal cell only found in the CT scan for recurrent chronic rhinosinusitis as one who had a diseased sphenoethmoidal cell. A diagnosis is made based on the consensus between Huang (otolaryngologist) and Lee (radiologist). Huang performed ESS following the standard routine to remove the disease in air cells. We defined visual loss as same-side visual acuity of or worse than the level of discrimination of hand movement. We followed it at the clinic or by a phone call if the patient did not come back to the clinic between Mar. and Sep. 2017.

We reported the incidence of an existing sphenoethmoidal cell by location as the number of existing sphenoethmoidal cell divided by the number of individuals. With the consideration of the losses of follow-up, we reported *the cumulative incidence* [17] of the diseased sphenoethmoidal cell by the following equation:

(1) $$\begin{array}{c} i=\frac{d}{p-0.5*l} \end{array}$$

where *i* is the cumulative incidence, *d* is the number of diseased sphenoethmoidal cells, *p* is the number of total individuals, and *l* is the number of those that did not have a diseased sphenoethmoidal cell in the available CT scans but lost to follow-up. The follow-up time since the first diagnosis of the diseased sphenoethmoidal cell was summarized. The calculations were performed in Matlab 2017a (MathWorks, Natick, MA).

## 3. Results

**Figure 1:**
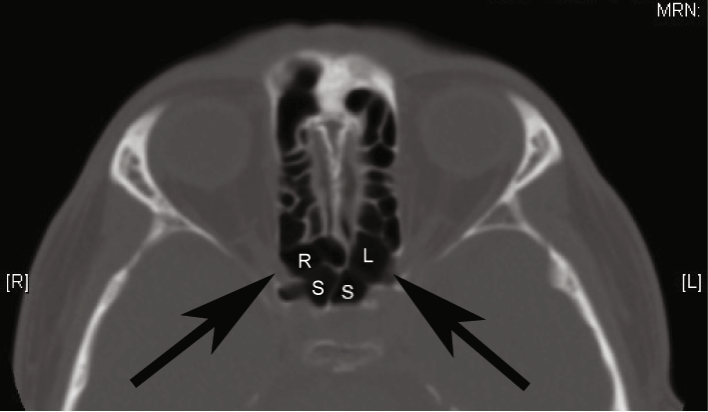
An example of bilateral sphenoethmoidal cells that meet the criteria of more than or equal to 5 mm posterior or superolateral extension. R: right sphenoethmoidal cell; L: left sphenoethmoidal cell; S: sphenoid sinus; arrow: optic canal.

**Figure 2:**
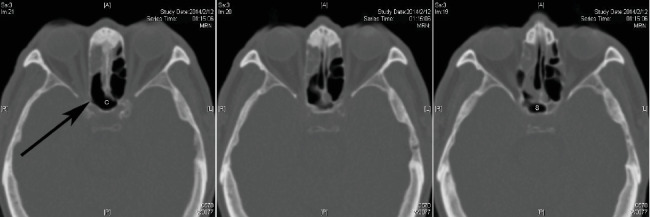
Three consecutive up-down CT images of an example of central sphenoethmoidal cell (C) that lies superiorly, extends more than 5 mm to the sphenoid sinus (S), and contacts right optic canal (arrow).

We enrolled 216 patients who underwent an ESS in the study. The mean age was 51 years (on the operation date) with a standard deviation of 16.7 years. One hundred and thirteen (52.3%) patients had at least one sphenoethmoidal cell. Eighty-five (39.4%) patients had a sphenoethmoidal cell on the right and 86 (39.8%) patients had one on the left. Two patients had a central sphenoethmoidal cell, which accounts for 0.9% of patients.  Figure 1 presents a case with bilateral sphenoethmoidal cells.  Figure 2 presents images of one patient with a central sphenoethmoidal cell.

**Figure 3:**
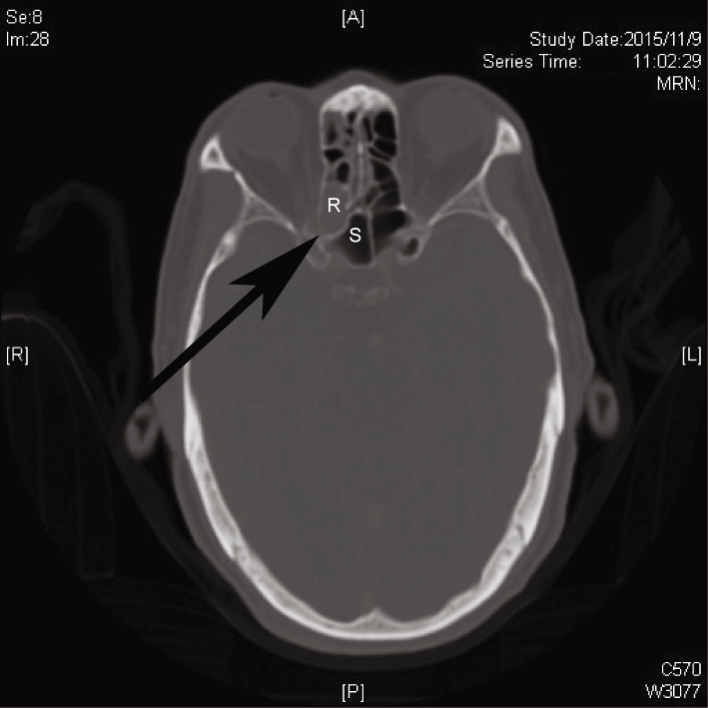
An example of right diseased sphenoethmoidal cell (R). S: sphenoid sinus; arrow: optic canal.

Opacity including sinusitis or mucocele was present in 23 diseased sphenoethmoidal cells on the right (23/85), 26 on the left (26/86), and 0 in the center. Thirty-two patients developed at least one diseased sphenoethmoidal cell.  Figure 3 presents a right diseased sphenoethmoidal cell. Ten out of the 32 patients were lost to follow-up. The cumulative incidence of a diseased sphenoethmoidal cell in patients with at least one existing sphenoethmoidal cell was 28.4% and in all patients 14.8%.

**Figure 4:**
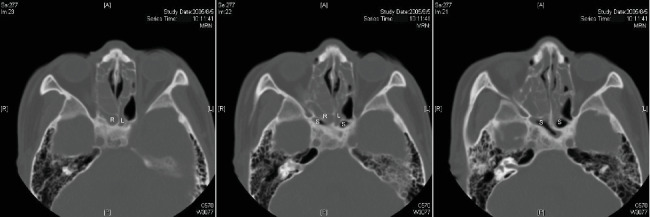
Initial presentation of the bilateral diseased sphenoethmoidal cells in 2005. Consecutive CT images of the patient who developed visual loss. R: right sphenoethmoidal cell; L: left sphenoethmoidal cell; S: sphenoid sinus.

**Figure 5:**
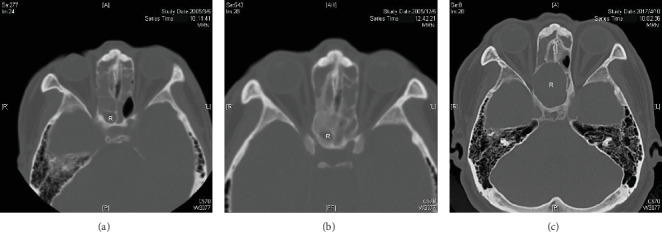
Progression of the right diseased sphenoethmoidal cell of the patient who developed visual loss in similar cuts of CT images in Aug. 2005 (a), Dec. 2005 (b), and Apr. 2017 (c). R: right sphenoethmoidal cell.

Among the 32 patients who developed at least one diseased sphenoethmoidal cell, one patient's CT showed bilateral opaque sphenoethmoidal cells (Figure 4) when he underwent his initial ESS in 2005. Twelve years (4412 days after the first CT) later, he presented to the hospital with acute right visual loss to the acuity of discrimination of hand movement, which was caused by the progressing right diseased sphenoethmoidal cells (Figure 5). He was then transferred to the otolaryngology department 30 days after the onset of visual loss. We performed image-guided ESS 11 days later (41 days after the onset of visual loss). However, the visual acuity of his right eye improved only from discrimination of hand movement to finger count after the surgery. The surgery successfully drained out the mucocele inside the right diseased sphenoethmoidal cell. It eroded tegmen of the ethmoid sinus and lamina papyracea. We lost 10 out of the 32 patients to follow-up, and no other one in the rest of 22 developed visual loss (12 visited the clinic regularly, and the other 10 were reached by a telephone survey due to their unwillingness to come back). The follow-up time since the first CT ranged from 70 to 4799 days with the mean follow-up days of 1750.

## 4. Discussion

A lack of systemic studies in the literature addressed the progression from a diseased sphenoethmoidal cell to visual loss. As mentioned in Introduction, the progression to visual loss is affected by various factors such as characteristics and location of the disease (e.g., an isolated mucocele in a lateral [5–7] or central [8] sphenoethmoidal cell, polyps or sinusitis [12, 13], or a fungal infection [14]). Some authors (e.g., [11, 12]) suggested that diabetics present little or no nasal symptoms and may be related to acute visual loss. Some reports (e.g., [18, 19]) proposed that tooth extraction and fracture of the sinus wall, permitting passage of pathogens, in an immunocompromised patient can lead to acute visual impairment because of orbital apex syndrome. Yet, as a descriptive study, a conclusion of what causes a diseased sphenoethmoidal cell or visual loss is misleading. With a relatively large sample number compared with the similar statistic reports in the literature, our results showed that 52.3% of ESS patients (*n* = 216) had at least one sphenoethmoidal cell, and 14.8% developed at least one diseased sphenoethmoidal cell. Among them, one patient was diagnosed with acute visual loss 12 years after the first CT, which suggests rare occurrence of acute severe visual loss caused by progressing diseased sphenoethmoidal cells in a short term.

The incidence of a sphenoethmoidal cell was reported from 8% to 60% [16, 20–25] varying with the definition and race. By our definition, 52.3% of 216 patients had at least one sphenoethmoidal cell.

The incidence of a central sphenoethmoidal cell [8–10] was rarely described, if ever, because of its anatomical relationship. Mucocele in a central sphenoethmoidal cell has been reported to cause simultaneous bilateral visual disturbance [8]. By our definition, the incidence of a central sphenoethmoidal cell in our study was 0.9% (2/216). We observed no diseased central sphenoethmoidal cell.

Disease abutting the optic nerve is one indication endorsed by the American Academy of Otolaryngology-Head and Neck Surgery [26] for the intraoperative use of image-guided surgery in appropriately select cases to assist the surgeon in clarifying complex anatomy during sinus and skull base surgery. The accuracy achieved by commercially available image-guided surgery systems can be less than or equal to 1.5 mm [27]. It is believed ethically impossible to corroborate the benefits of image-guided surgery with Level 1 evidence [26] for studies in the literature because of limitations of the study design [28]. Most surgeons believe that the image-guided surgery system is an important tool in minimizing the major complications such as visual loss [26, 28], as studies had showed a trend to fewer major complications with image-guided surgery systems (e.g., [29, 30]). The use of intraoperative surgical navigation for endoscopic sinus surgery could be considered for patients with a sphenoethmoidal cell.

## 5. Conclusions

Diseased sphenoethmoidal cells are associated with the risk of visual loss. Our results suggest that, even though a diseased sphenoethmoidal cell is frequent in people subjected to ESS, vision loss is infrequent among people with a diseased sphenoethmoidal cell. As over half of the patients have a sphenoethmoidal cell, we suggest preoperative and intraoperative identification of any sphenoethmoidal cells.

## Data Availability

Supporting images are all listed in the figures. Raw data of the subjects can be found in Supplementary Materials (available here).

## Supplementary Materials

Supplementary Materials Raw data of the subjects.
